# Imaging muscle as a potential biomarker of denervation in motor neuron disease

**DOI:** 10.1136/jnnp-2017-316744

**Published:** 2017-10-31

**Authors:** Thomas M Jenkins, James J P Alix, Charlotte David, Eilish Pearson, D Ganesh Rao, Nigel Hoggard, Eoghan O’Brien, Kathleen Baster, Michael Bradburn, Julia Bigley, Christopher J McDermott, Iain D Wilkinson, Pamela J Shaw

**Affiliations:** 1 Sheffield Institute for Translational Neuroscience, University of Sheffield, Sheffield, UK; 2 Department of Neurology, Sheffield Teaching Hospitals NHS Foundation Trust, Sheffield, UK; 3 Department of Neurophysiology, Sheffield Teaching Hospitals NHS Foundation Trust, Sheffield, UK; 4 Academic Unit of Radiology, University of Sheffield, Sheffield, UK; 5 Statistical Services Unit, School of Mathematics and Statistics, University of Sheffield, Sheffield, UK; 6 Clinical Trials Research Unit, School of Health and Related Research, University of Sheffield, Sheffield, UK

## Abstract

**Objective:**

To assess clinical, electrophysiological and whole-body muscle MRI measurements of progression in patients with motor neuron disease (MND), as tools for future clinical trials, and to probe pathophysiological mechanisms in vivo.

**Methods:**

A prospective, longitudinal, observational, clinicoelectrophysiological and radiological cohort study was performed. Twenty-nine patients with MND and 22 age-matched and gender-matched healthy controls were assessed with clinical measures, electrophysiological motor unit number index (MUNIX) and T2-weighted whole-body muscle MRI, at first clinical presentation and 4 months later. Between-group differences and associations were assessed using age-adjusted and gender-adjusted multivariable regression models. Within-subject longitudinal changes were assessed using paired t-tests. Patterns of disease spread were modelled using mixed-effects multivariable regression, assessing associations between muscle relative T2 signal and anatomical adjacency to site of clinical onset.

**Results:**

Patients with MND had 30% higher relative T2 muscle signal than controls at baseline (all regions mean, 95% CI 15% to 45%, p<0.001). Higher T2 signal was associated with greater overall disability (coefficient −0.009, 95% CI −0.017 to –0.001, p=0.023) and with clinical weakness and lower MUNIX in multiple individual muscles. Relative T2 signal in bilateral tibialis anterior increased over 4 months in patients with MND (right: 10.2%, 95% CI 2.0% to 18.4%, p=0.017; left: 14.1%, 95% CI 3.4% to 24.9%, p=0.013). Anatomically, contiguous disease spread on MRI was not apparent in this model.

**Conclusions:**

Whole-body muscle MRI offers a new approach to objective assessment of denervation over short timescales in MND and enables investigation of patterns of disease spread in vivo. Muscles inaccessible to conventional clinical and electrophysiological assessment may be investigated using this methodology.

## Introduction

A major barrier to effective therapeutics in motor neuron disease/amyotrophic lateral sclerosis (MND/ALS) is the lack of an objective measure of disease progression over short timescales that minimise expense in the context of clinical trials.^1^ A recent systematic review of MRI in MND reported 116 clinical studies, all of which investigated the central nervous system (CNS).^2^ Muscle denervation is a major clinical feature of MND and results in fluid shifts detectable as altered T2 signal characteristics^3^ that, in contrast to clinical assessments, are independent of participant’s effort and premorbid abilities. Whole-body MRI now enables sampling of multiple body regions during a single examination within acceptable timescales, including muscles inaccessible to conventional clinical or electrophysiological assessment. However, very few previous muscle MRI studies in MND exist,^4–7^ and none has assessed multiple body regions.

This is the first study to apply whole-body muscle MRI to patients with MND, who also underwent detailed longitudinal, clinical and electrophysiological phenotyping. The objective was to address the problem of anatomical phenotypic heterogeneity, which has represented a barrier to developing effective CNS imaging biomarkers, by adopting a muscle sampling methodology analogous to diagnostic El Escorial criteria.^8^ We hypothesised that (1) patients with MND would exhibit higher relative muscle T2 signal than healthy controls; (2) high relative muscle T2 signal would correlate with clinical weakness and lower electrophysiological motor unit number indices; (3) relative muscle T2 signal in patients with MND, but not controls, would increase over time and (4) changes in MRI parameters could be applied to probe pathophysiological mechanisms in vivo to model whether disease spread was anatomically contiguous.

## Patients and methods

### Study population and data collection

This was a prospective, longitudinal, observational cohort study. Consecutive patients were recruited at first presentation to the tertiary referral neuromuscular clinic at the Royal Hallamshire Hospital, Sheffield, UK. Inclusion criteria were age 18 years or over, a clinical diagnosis of MND fulfilling clinically possible, probable or definite revised El Escorial criteria^8^ or progressive muscular atrophy (defined as a pure lower motor neuron syndrome at study entry). Exclusion criteria were inability to give informed consent, a contraindication to MRI, coexistent neuromuscular disease, pregnancy or respiratory failure impairing ability to lie flat in the scanner. Healthy age-matched and sex-matched volunteers were recruited from spouses and friends of patients, via advertisement, and from clinical and research staff. The primary outcome measure was comparison of relative T2 signal on MRI between groups. In order to satisfy the requirement of at least 10–20 observations per degree of freedom for the linear regression models^9^ (with age and gender as predictors), a minimum sample size of 30–60 observations was required.

### Clinical, electrophysiological and neuroimaging assessments and analysis

Patients underwent clinical assessments and all participants underwent electrophysiological and imaging assessment at baseline and 4 months, on the same day. Four months were chosen pragmatically as a minimal interval to observe clinical change and minimise cohort attrition and informed by the only previous similar study.^5^ Age, gender, date and site of onset of symptomatic weakness were recorded. Patients completed the revised amyotrophic lateral sclerosis functional rating scale-revised (ALSFRS-R),^10^ a 48-point score, with lower scores indicating greater impact of MND on daily function. The following muscle groups were assessed and graded from 0 to 5 on the Medical Research Council (MRC) scale^11^ bilaterally: shoulder abductors, elbow flexors and extensors, wrist extensors and flexors, abductor pollicis brevis, hip flexors, knee flexors and extensors, ankle dorsiflexors, neck flexors and neck extensors, resulting in a maximum score of 110 with lower scores indicating greater weakness. Handheld dynamometry was performed in ankle dorsiflexors, after validation in a practice cohort, according to the established protocols.^12^ All participants were weighed on the same scales at each visit.

At each visit, maximum compound muscle action potential (CMAP) and motor unit number index (MUNIX) estimates^13^ were obtained from the least clinically affected side (to avoid ‘floor’ effects) in biceps and tibialis anterior (TA), using a Dantec Keypoint electromyography machine (Natus Medical, Pleasanton, California, USA), following internationally agreed protocols.^14^ Motor unit size (MUSIX) parameters were calculated as the ratio of CMAP to MUNIX; higher scores are associated with reinnervation.^15^

MRI was performed at 3 T (Ingenia 3.0T, Philips Healthcare, Best, the Netherlands). T2-weighted fast spin echo (repetition time (TR)=1107 ms, effective echo time (TE)=80 ms, interpolated voxel size=0.78×0.78×5 mm^3^) whole-body images were acquired in the coronal plane at six anatomical stations (Philips Healthcare). Acquisition time was 55 s per station and approximately 6 min total scan time (excluding localisers and breath-holds during thoracic and abdominal acquisitions).

Regions of interest were contoured manually according to the standardised anatomical landmarks (see online supplementary file 1) on single unprocessed coronal slices, in line with previous studies,^16^ using a semiautomated spline function provided in the system manufacturer’s online workstation (Extended MR Workspace V.2.6.3.5, Philips Healthcare). Intrarater and inter-rater reproducibility was first confirmed by two independent raters, who achieved coefficients of variation <5% for all regions of interest on six practice datasets at least 24 hours apart. Final contouring was performed by a single blinded operator (TMJ) and mean T2 signal estimates were derived for the tongue, right and left biceps, right and left thoracic paraspinals, right and left anterolateral leg compartment encompassing TA, second cervical vertebral body, right humerus and right tibia. Individual anterolateral leg compartment muscle boundaries are not readily distinguishable on coronal MR^5^ but TA predominates and is used for brevity for the remainder of this manuscript. Muscle region of interest T2 values were expressed relative to those obtained from bone within the same station (tongue:second cervical vertebral body, biceps:humerus, paraspinals:humerus and TA:tibia). A mean paraspinal muscle estimate was used for all analyses except where otherwise specified, in order for the thoracic domain to be represented by a single parameter, in accordance with El Escorial criteria.^8^ All scans were reviewed by a consultant neuroradiologist (NH) and incidental findings reported back to study participants.

10.1136/jnnp-2017-316744.supp1 Supplementary file 1

### Statistical analysis

Statistical analysis was performed in Stata V.13.1 and SAS/STAT V.9.3.

#### Patients versus controls at baseline

Independent binomial rate score-based CIs were used to assess for between-group gender differences. For continuous variables, between-group differences were assessed using multiple linear regression, entering each clinical, electrophysiological and MRI measure of interest, in turn, as response variables and patient/control group indicator, age and gender as predictor variables. For electrophysiological variables, the side tested was entered as an additional predictor.

#### Associations between muscle MRI and clinicoelectrophysiological measures

Associations between baseline clinical, electrophysiological and imaging parameters were assessed using regression models. Age, gender, days from symptom onset, ALSFRS-R and MRC summary score were each regressed, in turn, against whole-body relative T2 signal. Individual muscle MRC scores in biceps and TA and dynamometry in TA were each regressed, in turn, against the corresponding individual muscle relative T2 values. For electrophysiological parameters, the side tested was always regressed against the clinical or radiological parameter of corresponding laterality, entering the side tested as an additional predictor variable. Site of onset (leg, arm or bulbar) was specified as a binary variable and regressed against relative T2 signal from the corresponding anatomical region. Age and gender were entered into all models as additional predictor variables. Parameter estimates were reported for unadjusted models, and p values reported for both unadjusted and adjusted models.

Associations between each of the clinical parameters and MUNIX were also assessed, in turn, using the same methodology, always entering the side tested as an additional predictor variable.

#### Longitudinal changes

Within-group differences between time points were reported using two-tailed paired t-tests. The percentage of patients with a change in clinical and radiological measures was reported. To assess whether radiological changes were only found in muscles becoming clinically weak, correlation analysis assessing associations between changes in radiological parameters and changes in clinical parameters was performed. The percentage of slow clinical progressors, defined as a change in ALSFRS-R of one point or less, exhibiting radiological changes was reported. The responsiveness of each clinical, electrophysiological and radiological measure to change was assessed using standardised response means (mean/SD of measured change).^16^

#### Modelling disease spread

Anatomical patterns of disease spread from site of clinical onset in patients with MND were investigated by first calculating estimates of relative T2 signal change in each muscle, expressed as a ratio of 4 months to baseline measurements, natural log transformed, for each of the body regions, in turn: tongue, right biceps, left biceps, right thoracic paraspinal, left thoracic paraspinal, right TA and left TA. A distance variable was then created for each muscle in each patient, representing step changes in anatomical adjacency to site of clinical onset, at the level of the anterior horn cells supplying each muscle. The muscle of clinical onset (which was tongue, right arm, left arm, right leg, left leg, both arms or both legs) was allocated zero. Adjacent anatomical regions to the site of clinical onset were allocated one, considering both ‘horizontal’ anatomical organisation (eg, right leg to left leg) and ‘vertical’ organisation (ie, cranial to caudal along the neuroaxis, eg, right arm to right thoracic paraspinal or right thoracic paraspinal to right leg). If regions were two steps apart, they were allocated two (eg, tongue and right paraspinal). If regions were three steps apart, they were allocated three (eg, right arm and left leg). This distance variable was entered as an ordinal predictor into a mixed effects linear regression model with change in relative muscle T2 signal as the response variable, adjusting for baseline T2 muscle signal at baseline and including subject as a random effect.

#### Muscle area

A post hoc analysis was performed to assess differences in area for each muscle assessed. Intrarater and inter-rater reproducibility <5% was first achieved and then the entire muscle contoured. Baseline differences between patients and controls and longitudinal changes over time were assessed, using the same statistical methodology applied to investigate relative T2 signal, described in preceding sections.

For all analyses, statistical significance was reported at p<0.05 and a false discovery rate approach was used to correct for multiple comparisons.^17^

## Results

**Figure 1:**
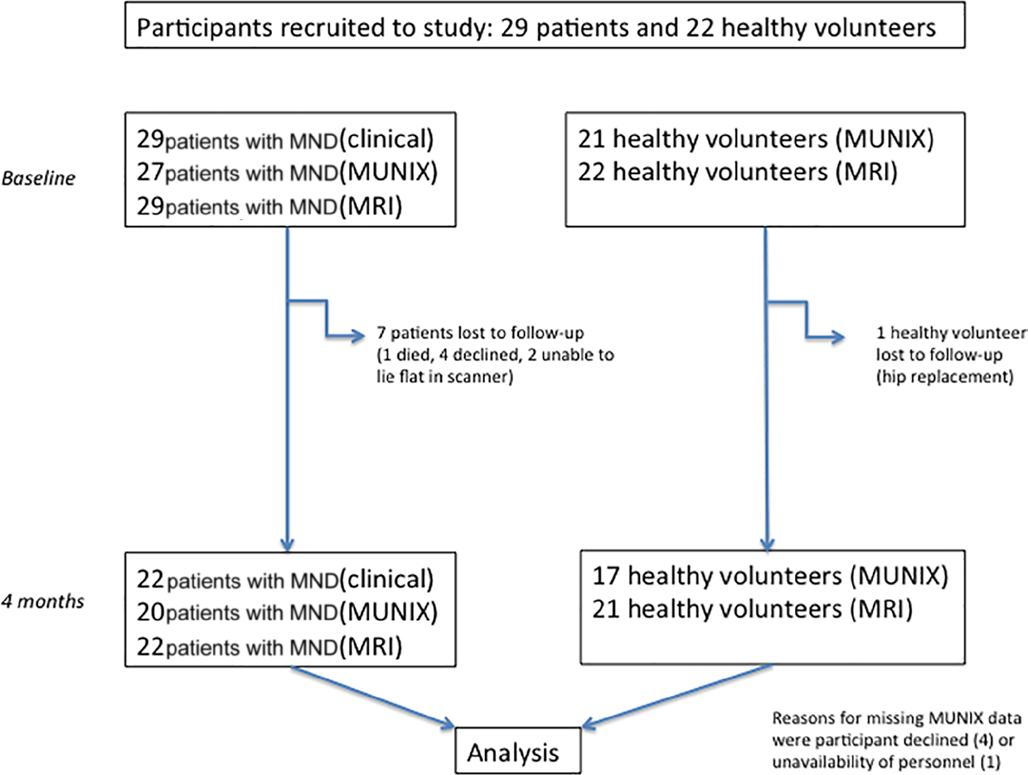
Flow chart of patients’ assessment and dropout. MND, motor neuron disease; MUNIX, motor unit number index.

Twenty-nine patients with MND and 22 healthy volunteers were recruited between October 2013 and May 2016 (figure 1). Groups were well matched for age, gender and weight (table 1). Twenty-six patients had ALS (six clinically definite, 12 clinically probable and eight clinically possible) and three had progressive muscular atrophy. Limb onset occurred in 25 patients (five right arm, seven left arm, two both arms, four right leg, five left leg and two both legs) and bulbar onset in four patients. Patients were recruited at a relatively early stage of disease (mean ALSFRS-R score 40, mean MRC summary score 100/110 and median duration of symptoms 15 months). Patients’ assessments were repeated after a mean of 133 days (95% CI 125 to 142 days) and controls 206 days (95% CI 172 to 239 days). Incidental liver cysts were found in 8%, Baker’s cysts in 6% and joint effusions in 6% of participants. All liver cysts were benign on subsequent ultrasound imaging. The only clinically relevant finding was a single patient with a leaking silicone breast implant. The patients’ clinical course was consistent with MND in all cases.

**Table 1:** Demographic, clinical, electrophysiological and neuroimaging baseline characteristics

	Patients (n=29)	Controls (n=22)	Difference, patients >controls (95% CI)	P value
Mean age, years (SD)	57.1 (13.5)	54.2 (15.7)	3.0 (−5.3 to 11.1)	0.474
Gender	7 (24.1%) females	9 (40.9%) females	−16.8% (−42.2% to 9.3%)	0.207
Mean weight, kg (SD)	77.7 (15.3)	75.0 (15.1)	2.7 (−6.1 to 11.4)	0.540 (0.746)
Median weeks from symptom onset (range)	66 (25–265)			
Mean MRC score (SD)	100/110 (10.6)			
Mean ALSFRS-R (SD)	40/48 (4.5)			
**Mean MUNIX biceps (SD)**	**129.3 (53.9)**	**200.2 (85.8)**	**−71.0 (−112.6 to –29.3)**	**0.001 (<0.001)**
**Mean CMAP biceps, mV (SD)**	**5.5 (2.2)**	**7.9 (3.4)**	**−2.3 (−4.0 to –0.7)**	**0.007 (0.002)**
Mean MUSIX biceps (SD)	41.4 (9.6)	39.6 (4.1)	1.7 (−2.9 to 6.3)	0.459 (0.990)
**Mean MUNIX TA (SD)**	**83.5 (48.6)**	**139.3 (32.2)**	**−55.8 (−80.6 to –30.9)**	**<0.001 (<0.001)**
**Mean CMAP TA, mV (SD)**	**4.2 (1.9)**	**5.4 (3.7)**	**−1.2 (−2.1 to –0.3)**	**0.002 (0.002)**
**Mean MUSIX TA (SD)**	**56.4 (27.3)**	**39.7 (6.4)**	**16.7 (4.5 to 29.0)**	**0.009 (0.016)**
**MRI relative T2 signal summary score all regions (SD)**	**0.39 (0.10)**	**0.30 (0.04)**	**30.3% (15.0% to 45.5%)**	**<0.001 (<0.001)**
MRI relative T2 signal tongue (SD)	0.69 (0.13)	0.64 (0.09)	8.3% (−1.6% to 18.2%)	0.098 (0.082)
**MRI relative T2 signal right biceps (SD)**	**0.38 (0.10)**	**0.30 (0.06)**	**25.2% (9.2% to 41.1%)**	**0.003 (0.003)**
**MRI relative T2 signal left biceps (SD)**	**0.19 (0.15)**	**0.12 (0.04)**	**62.6% (7.1% to 118.1%)**	**0.028 (0.034)**
**MRI relative T2 signal thoracic paraspinals (SD)**	**0.45 (0.17)**	**0.31 (0.09)**	**42.4% (16.9% to 68.0%)**	**0.002 (0.006)**
**MRI relative T2 signal right TA (SD)**	**0.38 (0.16)**	**0.27 (0.05)**	**37.6% (12.4% to 62.8%)**	**0.004 (0.011)**
**MRI relative T2 signal left TA (SD)**	**0.24 (0.15)**	**0.14 (0.05)**	**73.3% (26.4% to 120.1%)**	**0.003 (0.005)**

Parameter estimates refer to unadjusted differences between patients and controls, with 95% CI in parentheses. Pvalues refer to differences between patients and controls unadjusted and, in parentheses, adjusted for age and gender. Relative T2 signal differences are reported as percentage differences in patients with motor neuron disease relative to controls. Significant results after correction for multiple comparisons are highlighted in bold.

ALSFRS-R, amyotrophic lateral sclerosis functional rating scale-revised; CMAP, compound muscle action potential; MRC, Medical Research Council; MUNIX, motor unit number index; MUSIX, motor unit size index; TA, tibialis anterior.

### Patients versus controls at baseline

**Figure 2:**
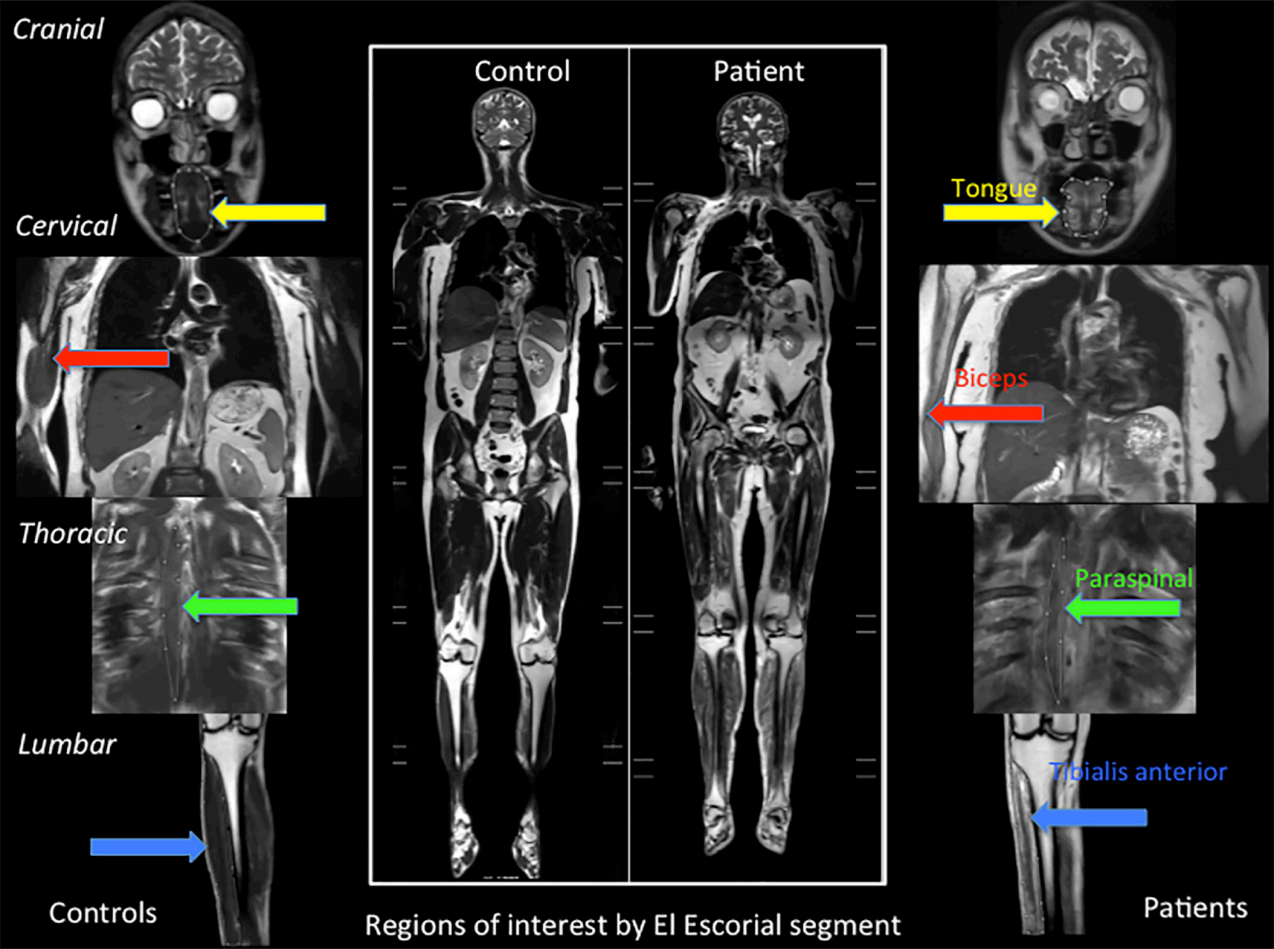
Baseline data in patients and controls. The central panel shows illustrative central coronal slices from whole-body T2-weighted datasets obtained in a healthy control (left) and a patient with MND (right). Extracted regions of interest are demonstrated in controls on the left and a patient with MND on the right in the tongue (yellow arrows), right biceps (red arrows), right thoracic paraspinal (green arrows) and right anterolateral leg compartment encompassing tibialis anterior (blue arrows).

**Figure 3:**
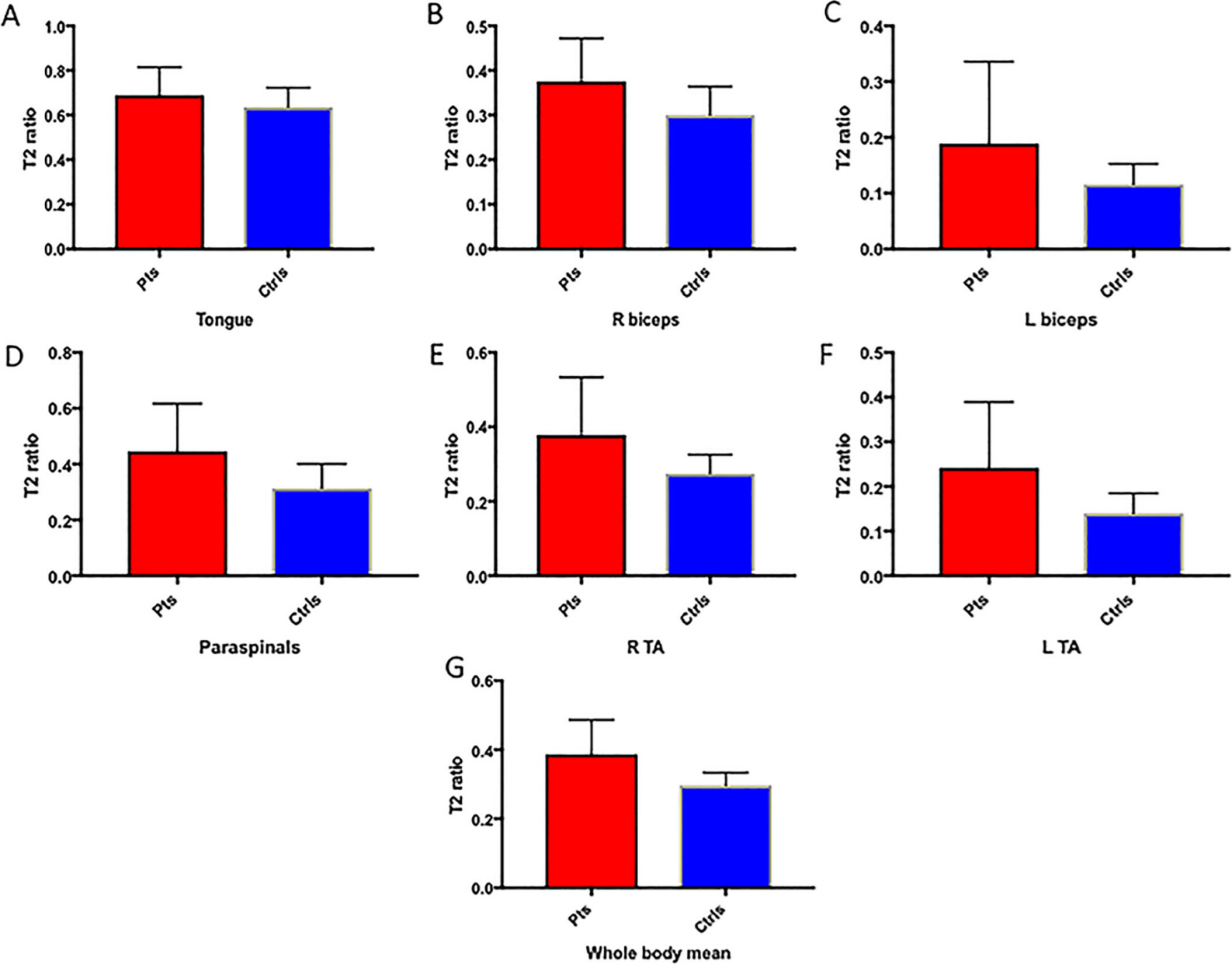
Box plots illustrating means and SD of relative T2 signal in patients with MND (red) and controls (blue) in the (A) tongue, (B) right biceps, (C) left biceps, (D) thoracic paraspinals, (E) right TA, (F) left TA and (G) mean all regions. Ctrls, controls; L, left; Pts, patients; R, right; TA, tibialis anterior.

On baseline electrophysiology, patients with MND had lower MUNIX and CMAPs than controls in both TA and biceps (table 1). MUSIX was higher in patients’ TA. At baseline MRI assessment, patients had higher relative T2 signal than controls in all muscles except the tongue, in which there was a trend towards increased relative T2 signal in the patient group (table 1 and figures 2 and 3).

### Associations between muscle MRI and clinicoelectrophysiological measures

Higher relative muscle T2 signal was associated with greater weakness in each of the tested muscles on MRC scoring, except right biceps. Higher relative T2 signal was associated with greater weakness measured using dynamometry in TA bilaterally (table 2). Higher relative T2 signal was associated with lower MUNIX and CMAPs. Leg-onset disease was associated with higher relative T2 in TA (regression coefficients: right 0.14 units (95% CI 0.03 to 0.25, p=0.013); left 0.14 units (95% CI 0.03 to 0.24, p=0.012)), but there were no associations in biceps or the tongue for arm and bulbar-onset disease, respectively.

**Table 2:** Associations between muscle relative T2 signal and corresponding regional, clinical and electrophysiological measures

Parameter associated with relative T2 signal	Regression coefficient (95% CI)	P value
Age	0.001 (−0.002 to 0.004)	0.395
Gender	0.014 (−0.076 to 0.104)	0.749
Days from symptom onset	0.00004 (−0.00004 to 0.00012)	0.345 (0.227)
**ALSFRS-R**	**−0.009 (−0.017 to –0.001)**	**0.023 (0.022)**
MRC summary score	−0.003 (−0.0068 to 0.0001)	0.060 (0.078)
MRC score right biceps	−0.017 (−0.064 to 0.030)	0.469 (0.794)
**MRC score left biceps**	**−0.079 (−0.147 to –0.011)**	**0.024 (0.037)**
**MRC score right TA**	**−0.107 (−0.142 to –0.071)**	**<0.001 (<0.001)**
**MRC score left TA**	**−0.069 (−0.091 to –0.046)**	**<0.001 (<0.001)**
**Dynamometry right TA**	**−0.005 (-0.007 to –0.002)**	**<0.001 (<0.001)**
**Dynamometry left TA**	**−0.005 (−0.007 to –0.003)**	**<0.001 (<0.001)**
**MUNIX biceps**	**−0.001 (−0.0020 to –0.0002)**	**0.015 (0.077)**
**CMAP biceps**	**−0.027 (−0.049 to –0.005)**	**0.019 (0.092)**
MUSIX biceps	−0.0007 (−0.0063 to 0.0048)	0.783 (0.703)
**MUNIX TA**	**−0.0018 (−0.0029 to –0.0007)**	**0.003 (0.001)**
**CMAP TA**	**−0.055 (−0.082 to –0.028)**	**<0.001 (<0.001)**
MUSIX TA	−0.0008 (−0.0032 to 0.0015)	0.460 (0.509)

Parameter estimates refer to unadjusted coefficients derived from regression models, with 95% CI in parentheses. P values refer to regression models unadjusted and, in parentheses, adjusted for age and gender. Significant results after correction for multiple comparisons are highlighted in bold.

ALSFRS-R, amyotrophic lateral sclerosis functional rating scale-revised; CMAP, compound muscle action potential; MRC, Medical Research Council; MUNIX, motor unit number index; MUSIX, motor unit number size; TA, tibialis anterior.

Associations between MUNIX and clinical measures are reported in online supplementary table S1.

### Longitudinal changes

In healthy controls, there were no longitudinal changes in relative T2 signal or MUNIX in any muscle. Variability of relative T2 signal estimates within the control group is reported in online supplementary table S2.

**Figure 4:**
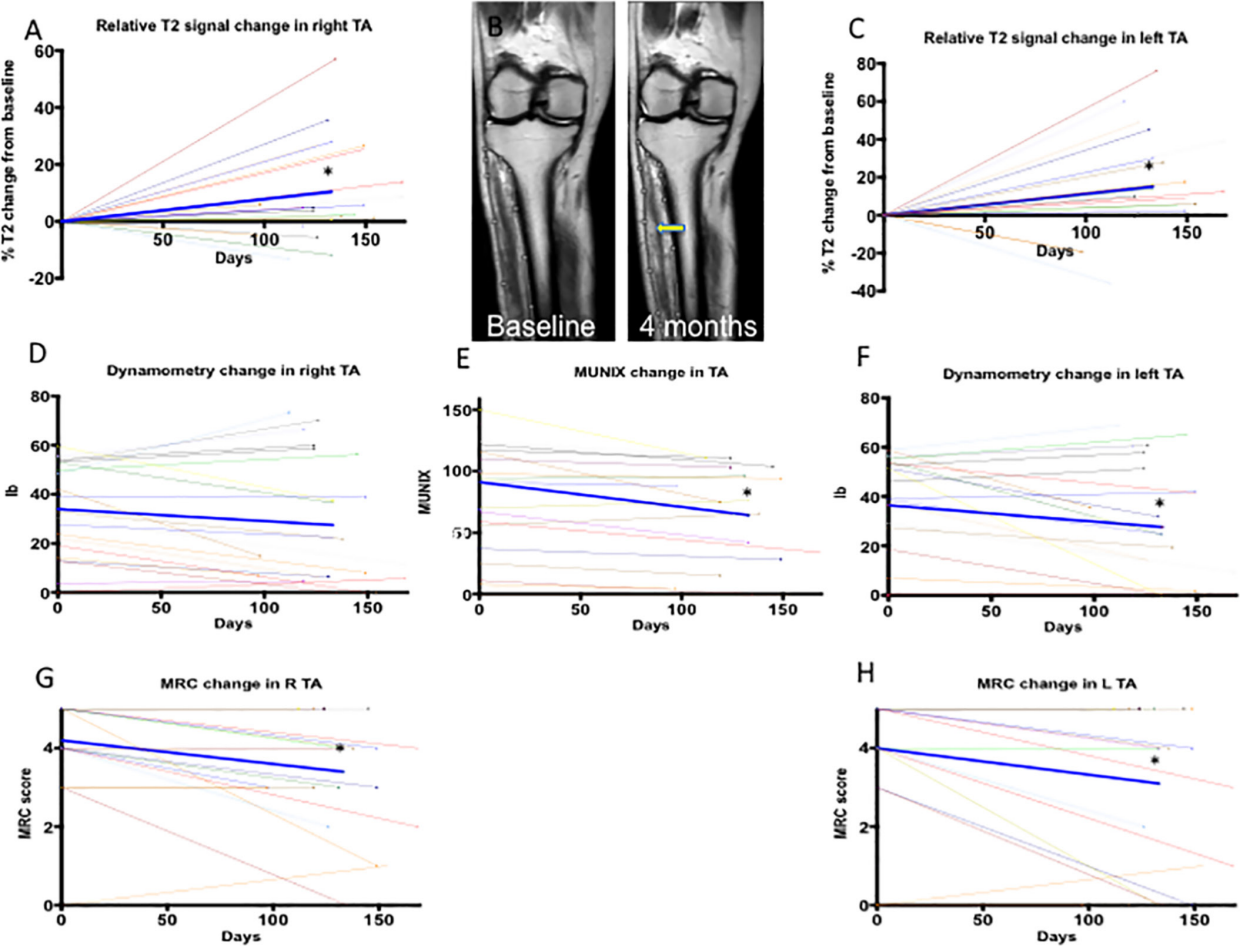
Graphs depict longitudinal changes in TA parameters: (A) percentage relative T2 signal on right, (B) coronal T2-weighted slices from a patient with motor neuron disease at baseline and 4 months illustrating an increase in relative T2 signal on the right (yellow arrow), (C) percentage relative T2 signal change on left, (D) dynamometry on right, (E) MUNIX tested, (F) dynamometry on left, (G) MRC score on right and (H) MRC score on left. The bold blue line represents mean change. Asterisks indicate statistically significant change on paired t-tests (p<0.05 corrected for multiple comparisons). MRC, Medical Research Council; MUNIX, motor unit number index; TA, tibialis anterior.

Longitudinal changes in clinical, electrophysiological and MRI parameters in patients with MND were evident (table 3 and figure 4). Clinical change was most evident in TA bilaterally. There was also a statistically significant decrease in MUNIX in TA. Relative T2 signal values increased significantly in both TA muscles in patients with MND with increases evident in 73% and 82% of patients in right and left TA, respectively. This contrasted with decreases in power, which were evident in 45% and 59% of patients when measured using dynamometry and 41% and 50% of patients when measured using MRC scores in right and left TA, respectively.

**Table 3:** Longitudinal change in clinical, neurophysiological and MRI parameters in patients with motor neuron disease between baseline and 4 months

Parameter	Change from baseline to 4 months (95% CI)	t Value (df)	P value	SRM
**ALSFRS-R score (% change from baseline)**	**−3.5 (−5.2 to –1.8); −7.3% (−10.8% to −3.8%)**	**−4.2 (21)**	**<0.001**	**−0.90**
**Total MRC score**	**−8.4 (−11.1 to –5.8)**	**−6.6 (21)**	**<0.001**	**−1.40**
MRC score right biceps	−0.2 (−0.528 to 0.007)	−2.0 (21)	0.057	−0.43
**MRC score left biceps**	**−0.4 (−0.7 to –0.1)**	**−2.6 (21)**	**0.017**	**−0.55**
**MRC score right TA**	**−0.8 (−1.3 to –0.3)**	**−3.1 (21)**	**0.005**	**−0.67**
**MRC score left TA**	**−0.9 (−1.5 to –0.3)**	**−3.0 (21)**	**0.007**	**−0.64**
Dynamometry right TA (lb)	−4.6 (−10.6 to 1.4)	−1.6 (21)	0.128	−0.34
**Dynamometry left TA (lb)**	**−8.3 (−15.1 to –1.5)**	**−2.5 (21)**	**0.019**	**−0.54**
Weight (kg)	−0.7 (−2.0 to 0.7)	−1.1 (17)	0.302	−0.25
MUNIX biceps	−17.4 (−35.0 to 0.1)	−2.1 (18)	0.052	−0.48
**CMAP biceps**	**−0.7 (−1.3 to –0.2)**	**−2.7 (18)**	**0.016**	**−0.61**
MUSIX biceps	2.4 (−2.6 to 7.4)	1.0 (18)	0.326	0.23
**MUNIX TA**	**−11.0 (−17.9 to –4.0)**	**−3.3 (17)**	**0.004**	**−0.78**
CMAP TA	−0.2 (−0.7 to 0.2)	−1.2 (17)	0.239	−0.29
MUSIX TA	4.1 (−6.5 to 14.7)	0.8 (17)	0.428	0.19
T2 signal whole-body average (% change from baseline)	−0.01 (−0.04 to 0.02); −2.4% (−5.6% to 10.3%)	0.6 (21)	0.542	0.13
T2 signal from muscle region of clinical onset (% change)	0.02 (−0.02 to 0.05); 4.7% (−0.5% to 14.4%)	1.0 (21)	0.322	0.22
T2 signal tongue (% change)	−0.02 (−0.07 to 0.02); −3.4% (−9.9% to 3.0%)	−1.1 (21)	0.282	−0.24
T2 signal right biceps (% change)	−0.01 (−0.07 to 0.04); −3.1% (−17.5% to 11.3%)	−0.5 (21)	0.656	−0.10
T2 signal left biceps (% change)	−0.05 (−0.10 to 0.01); −23.1% (−52.7% to 6.6%)	−1.6 (21)	0.121	−0.34
T2 signal thoracic paraspinals (% change)	0.05 (−0.10, 0.01); −10.6% (−23.1% to 1.8%)	−1.8 (21)	0.091	−0.38
**T2 signal right TA (% change)**	**0.04 (0.01 to 0.07); 10.2% (2.0% to 18.4%)**	**2.6 (21)**	**0.017**	**0.55**
**T2 signal left TA (% change)**	**0.03 (0.01 to 0.06); 14.1% (3.4% to 24.9%)**	**2.7 (21)**	**0.013**	**0.58**

Parameter estimates and P values refer to paired t-tests. Significant changes after correction for multiple comparisons are highlighted in bold.

ALSFRS-R, amyotrophic lateral sclerosis functional rating scale-revised; CMAP, compound muscle action potential; MRC, Medical Research Council; MUNIX, motor unit number index; MUSIX, motor unit number size; SRM, standardised response mean; TA, tibialis anterior.

Moderate associations were found between increases in relative T2 signal and decreases in MRC scores in TA (right r=−0.63, p=0.002; left r=−0.50, p=0.017). No significant associations were found between changes in relative T2 signal and changes in dynamometry in TA.

Six of the 22 patients with MND with longitudinal follow-up were designated as slow progressors; two patients decreased by one point, two patients did not change and two patients exhibited an increase of one point in ALSFRS-R. In this subgroup, mean relative T2 signal increases of 8.1% and 18.9% were seen in right and left TA, respectively (with increases seen in 4/6 individuals on each side).

Responsiveness was high for global clinical measures and moderate for regional clinical measures, electrophysiological and radiological change variables.

A post hoc analysis demonstrated that all results reported in tables 1–3 retained significance when the three patients with progressive muscular atrophy were excluded.

### Modelling disease spread

There was no association between muscle relative T2 signal change and distance from site of clinical onset.

### Muscle area

There were no differences in total or individual muscle area at baseline between patients with MND and controls, except in the thoracic paraspinals after correction for age and gender (see online supplementary table S3). There was no evidence of atrophy in any muscles between baseline and 4 months.

## Discussion

The key results from this study are: (1) patients with MND exhibit higher relative T2 signal on muscle MRI than controls; (2) higher relative T2 signal in patients with MND is associated with clinical weakness and lower electrophysiological motor unit numbers in limb muscles; (3) relative T2 signal in bilateral TA increases over 4 months in patients with MND and (4) modelling pathophysiological mechanisms in vivo is feasible using this methodology, but no evidence for contiguous disease spread was demonstrated. We conclude that relative T2 signal in muscle reflects clinically relevant aspects of MND pathophysiology, and the ability to detect longitudinal changes in TA suggests that the technique has potential as an objective disease measure.

A biomarker to objectively measure progression over short timescales represents a key area of need in MND with important implications for future clinical trials.^18^ Existing trial outcome measures such as survival, respiratory failure, change in ALSFRS-R, manual muscle power or upper motor neuron scores^19^ have well-documented limitations.^20 21^ Responsiveness was higher for clinical measures than radiological and electrophysiological changes in our study, due to lower variance, but muscle MRI changes were evident in the subgroup of slow progressors. Moreover, a higher proportion of patients exhibited radiological change in TA than decreases in MRC scores or dynamometry, and correlation analysis suggested that clinical weakness and high relative T2 signal reflect complementary but distinct aspects of pathophysiology. While CNS biomarkers have been well investigated, peripheral biomarkers in MND are an exciting new research area. A recently published longitudinal study was the first to assess the peripheral nerves in MND using diffusion tensor imaging and demonstrated changes reflecting axonal degeneration that were associated with clinical and electrophysiological deterioration over 6 months.^22^ Electrical impedance myography also appears a promising technique,^23^ but muscle MRI remains largely unexplored. Early studies described qualitative morphological changes in the tongue in MND.^4 24^ A small study of the leg muscles identified T2 relaxation, but not quantitative T1 or volumetric, changes in a group of 11 patients, compared with eight controls, followed over 4 months.^5^ T2 signal change is correlated with changes in CMAPs and maximum voluntary isometric contraction. There were no further studies of muscle MRI in MND for 15 years, until our group reported a small pilot study, in which muscle atrophy lagged behind clinical change in four patients over 1-year follow-up.^25^ Two more recent studies were reported on patients with MND who had undergone brachial plexus imaging: the first reported abnormal appearances of arm muscles in 57%,^6^ and the second altered signal, graded qualitatively, in 70%, 43% and 30% of patients in supraspinatus, subscapularis and infraspinatus, respectively.^7^ No previous studies have applied a whole-body MRI methodology to study muscle changes in individuals with MND.

Following experimental denervation, reversible high T2 signal on muscle MRI is evident from 48 hours after injury,^26^ associated with an increased extracellular fluid space compartment^27 28^ and later with endomysial and perimysial fat deposition between 2 and 20 weeks.^29^ In clinical studies, MRI changes appear after 4 days;^30^ fatty changes predominate later.^31^ At the disease stage of our patients, it is likely that both fluid compartment shifts and sarcolemmal fat replacement related to denervation (evidenced by associations with lower MUNIX) are responsible for the observed relative T2 changes. However, as we did not obtain fat-quantification sequences, we cannot distinguish the relative contributions, which is a limitation of our study. In addition, T2 measurements were not derived using quantitative relaxometry because of prohibitive scan times for whole-body imaging in patients with MND. A recent study of patients with neuropathy/myopathy obtained quantitative T2, fat fraction and magnetisation transfer ratio measurements in 35 min for leg muscles alone,^16^compared with 6 min for whole-body sequences in our study (20 min total including set-up, localisers and breath-holds). Our semiquantitative T2 measurements were adjusted using adjacent bone references, which requires an assumption that bone signal remains constant. This may not always hold true, for example, in menstruation or osteoporosis, but these factors were not relevant in our study. Other limitations include potential region of interest sampling errors between baseline and follow-up, as automated segmentation of whole-body acquisitions remains challenging and final analysis was performed by a single rater, heterogeneity in disease course and longer follow-up intervals in controls compared with patients. Our patient cohort appears slightly younger with a slower rate of progression and lower prevalence of bulbar function than the MND population in general. Nonetheless, we appear to have captured a biologically feasible effect in a rapid, low burden and relatively low-cost assessment, with the advantage of comprehensive muscle sampling that a whole-body acquisition confers.

One such advantage is that mechanistic hypotheses can be tested. Clinical and pathological observations suggest that MND spread occurs in an anatomically contiguous manner,^32 33^ with prion-like behaviour in experimental models reported.^34^ We found no associations to support this hypothesis in our model. This could be because disease spread in our cohort was genuinely non-contiguous at spinal level, instead influenced by cortical contiguity (which was not assessed), or relate to methodological limitations, for example, differential sensitivity to detect disease effects in different muscles, non-linear progression patterns or insufficient statistical power. Future studies could investigate a greater number of muscles in larger cohorts or adopt non-linear modelling approaches.

In the present study, muscle abnormalities compared with controls, and clinicoradiological associations, were widespread and included muscles in which clinical and quantitative neurophysiological assessments are challenging, such as thoracic paraspinals. In contrast, longitudinal change was only evident in TA. Possible reasons include a high prevalence of limb-onset disease and more rapid rate of progression of weakness in TA compared with biceps, both evident in our cohort. TA may represent a good target in future longitudinal studies, regardless of site of disease onset. An advantage of whole-body acquisition is that other vulnerable muscles may be identified.

In summary, this is the first study to assess whole-body muscle MRI in patients with MND, and our findings of biologically feasible longitudinal changes are encouraging. Techniques to quantify upper motor neuron pathology exist, such as threshold tracking transcranial magnetic stimulation.^35 36^ As MND is a heterogeneous condition involving both upper and lower motor neuron dysfunction, future trials may benefit from a combined biomarker approach. Muscle MRI may quantify elements of lower motor neuron dysfunction over timescales as short as 4 months and identify promising candidate muscles to target with a formal quantitative T2 relaxometry approach.
