# Supplementary material for: Imaging muscle as a potential biomarker of denervation in motor neuron disease
Source: J Neurol Neurosurg Psychiatry. 2017 Oct 31;89(3):248–55. doi: 10.1136/jnnp-2017-316744 (PMC5869448; doi:10.1136/jnnp-2017-316744)
Supplement: Supplementary file 1 [file jnnp-2017-316744supp001.pdf]

Supplementary material

METHODS

Contouring protocol

Tongue: the most anterior coronal slice in which bilateral temporal lobes were visible was selected.

Second cervical vertebral body: the most anterior coronal slice incorporating the entire vertebral body was contoured.

Biceps: the most anterior coronal slice with maximal muscle coverage in the left-right axis was selected.

Humerus: the most anterior coronal slice incorporating the entire humeral head was contoured.

Thoracic paraspinals: the most anterior coronal slice with complete coverage was selected.

Antero-lateral leg compartment encompassing tibialis anterior: the most anterior coronal slice was selected, in which the full length of the anterolateral leg compartment and at least 2.5cm of muscle in right/left axis was visible.

Tibia: the most anterior coronal slice incorporating tibial tuberosity was selected.

RESULTS

Table S1 Associations between MUNIX and clinical scores

Parameter associated with MUNIX	Regression coefficient (95%CI)	p value
**MRC biceps**	**0.009 (0.004, 0.015)**	**0.002**
**MRC tibialis anterior**	**0.014 (0.006, 0.022)**	**0.001**
**Dynamometry tibialis anterior**	**0.023 (0.100, 0.367)**	**0.001**

Parameter estimates and p-values refer to associations derived from regression models unadjusted and, in parentheses, adjusted for age and gender. Significant results after correction for multiple comparisons are highlighted in bold. CI, confidence interval; MRC, Medical Research Council; MUNIX, motor unit number index; TA, tibialis anterior.

Table S2 Longitudinal variance in relative T2 signal in controls between baseline and four months

Muscle	Percentage T2 signal change at 4 months relative to baseline (95%CI)	p value
Whole-body average	-0.6% (-5.4%, 4.1%)	0.782
Tongue	2.0% (-3.1%, 7.1%)	0.426
Right biceps	-5.8% (-17.2%, 5.6%)	0.304
Left biceps	-4.3% (-22.5%, 13.9%)	0.629
Thoracic paraspinals	3.4% (-5.4%, 12.1%)	0.428
Right tibialis anterior	-1.7% (-6.9%, 3.5%)	0.502
Left tibialis anterior	-5.5% (-17.2%, 6.3%)	0.343

Parameter estimates and p values refer to paired t-tests. CI, confidence interval.

Table S3 Differences in muscle area at baseline between MND patients and controls

Muscle	Area (cm^2)		Difference patients>controls (95%CI)	p value
	Patients	Controls		
Total all regions mean (SD)	365.65 (129.97)	376.10 (136.37)	-10.46 (-86.53, 65.62)	0.783 (0.516)
Tongue mean (SD)	16.65 (2.75)	16.28 (3.49)	0.37 (-1.41, 2.14)	0.681 (0.649)
Right biceps mean (SD)	37.68 (12.95)	42.70 (13.30)	-5.02 (-12.52, 2.49)	0.185 (0.042)
Left biceps mean (SD)	43.79 (14.37)	43.95 (14.34)	-0.16 (-8.38, 8.06)	0.969 (0.419)
Thoracic paraspinals mean (SD)	15.65 (7.36)	21.80 (10.91)	-6.15 (-11.29, -1.01)	0.020 **(0.007)**
Right tibialis anterior mean (SD)	128.24 (67.30)	125.37 (61.95)	2.87 (-34.09, 39.84)	0.877 (0.980)
Left tibialis anterior mean (SD)	122.70 (63.51)	126.00 (55.45)	-3.30 (-37.49, 30.90)	0.847 (0.574)

Parameter estimates and p values refer to unadjusted differences between patients and controls, with 95% confidence intervals in parentheses. P values refer to differences between patients and controls unadjusted and, in parentheses, adjusted for age and gender. Significant results after adjusting for multiple comparisons are highlighted in bold. CI, confidence interval; cm, centimetre; SD, standard deviation.

There was no longitudinal volume loss over time in any muscle group.
